# Right Temporoparietal Junction Modulates In-Group Bias in Facial Emotional Mimicry: A tDCS Study

**DOI:** 10.3389/fnbeh.2020.00143

**Published:** 2020-08-28

**Authors:** Shenli Peng, BeiBei Kuang, Ping Hu

**Affiliations:** ^1^College of Education, Hunan Agricultural University, Changsha, China; ^2^The Institute for Mental Crisis Prevention and Intervention of College Students in Jiangsu Province, Nanjing Audit University, Nanjing, China; ^3^Department of Psychology, Renmin University of China, Beijing, China

**Keywords:** rTPJ, ethnic group membership, in-group bias, facial emotional mimicry, tDCS

## Abstract

The present study employs transcranial direct current stimulation (tDCS), a non-invasive brain stimulation technique, to explore the possible role of the right temporoparietal junction (rTPJ) in regulating in-group bias in facial emotional mimicry. Participants received either anodal or cathodal stimulation, or they were assigned to a sham condition. After that, they passively viewed a series of video clips depicting different emotions (happiness and anger) that were performed either by ethnic in-group or out-group models. The emotion-specific muscle activities, zygomatic major (ZM) and corrugator supercilii (CS) were recorded simultaneously as the index of facial emotional mimicry. The results first confirm the in-group bias in facial emotional mimicry in the sham condition, as shown in prior studies, though it only occurs in happy mimicry. Moreover, the in-group bias in facial emotional mimicry is modulated by the cortical excitability over the rTPJ, which may be attributed to the accompanied change of overlap of the mental representations of in-group and out-group. This study provides a close look at the neural underpinning of the modulation of facial emotional mimicry by group membership and highlights the role of rTPJ in on-line control of co-activated self and other representations in social cognition.

## Introduction

Mimicry occurs frequently in our daily life. Imagine you are listening to a friend of yours as she happily shares her summer vacation experience. You might not be aware that you are copying her facial expression unconsciously (e.g., the way she lifts the corners of her mouth or raises her eyebrows). People tend to mimic others’ nonverbal emotional expressions automatically in social interaction, which is termed emotional mimicry (Hess and Fischer, 2013; Hess et al., 2014). Emotional mimicry counts much in social life, as it can foster social bonding (Stel and Vonk, 2010), facilitate emotional understanding (Stel and van Knippenberg, 2008), enhance prosocial behavior (van Baaren et al., 2004), and strengthen group membership (Hess and Fischer, 2017).

Not all mimicry is equal. For example, one would never mimic his or her rival’s smile. Pain contagion occurs only between friends but not strangers (Martin et al., 2015). Empirical studies have confirmed this idea by showing that people mimicked more in-group (relative to out-group) emotions (Mondillon et al., 2007; Weisbuch and Ambady, 2008). Of particular interest, facial emotional mimicry, indexed by congruent facial muscle activities in response to the observed emotional expressions (Seibt et al., 2015; Wood et al., 2016), is modulated by group membership (Bourgeois and Hess, 2008; van der Schalk et al., 2011; Deng, 2018). For instance van der Schalk et al. (2011, study 1) manipulated the group membership with subject categories to explore whether it could modulate facial emotional mimicry. The authors recruited a group of psychology students to record their facial electromyographical (EMG) responses to the emotional photos performed by models labeled either as psychology students (in-group) or economics students (out-group). The results demonstrated an in-group superiority in mimicking angry and fearful emotional expressions. To summarize, Hess and Fischer (2013, 2014) proposed the Emotional Mimicry in Context (EMC) view, suggesting that emotional mimicry was not a simple motor or muscle activity convergence, but a complex process modulated by many contextual factors, i.e., group membership.

However, previous research concerning the effect of group membership on facial emotional mimicry remains inconsistent. In a replicated study of van der Schalk et al. (2011), Sachisthal et al. (2016) failed to reproduce the original outcomes. During a similar procedure, participants in Sachisthal et al. (2016) study viewed various emotional video clips performed by ethnic in-group and out-group members. The results revealed there was neither in-group nor out-group superiority in facial emotional mimicry across all emotions (e.g., anger/fear). Moreover, there was an increased mimicry to out-group angry faces compared to in-group angry faces (Rauchbauer et al., 2016). In terms of happy mimicry, previous findings are also perplexing. Some researchers have found participants’ mimicry to be equivalent between the in-group happiness and out-group happiness (Bourgeois and Hess, 2008; Ardizzi et al., 2014; Sachisthal et al., 2016), while others have shown that participants displayed stronger mimicry for in-group happiness than for out-group happiness (e.g., Weisbuch and Ambady, 2008; Deng, 2018; Peng et al., under review). In an unpublished thesis, Deng (2018) manipulated group membership with different ethnicities. A group of Chinese college students passively viewed a series of dynamic expressions, which were created from a set of pictures morphed between the neutral expression and either the happy or angry expression of the same face identity. Facial EMG activations were recorded simultaneously. Using the inclusion of others in the self scale (IOS, Aron et al., 1992), the author confirmed the manipulation of group membership by showing participants reported higher overlap between self and ingroup than the overlap between self and outgroup. Furthermore, the findings indicated that participants mimicked more in-group (vs. out-group) happiness, while there was no difference between the mimicry of in-group and out-group anger. To note, no significant mimicry-related EMG activations (relative to baseline) were found in *Deng’s study* for either in-group or out-group anger. This was in line with previous studies (e.g., Rymarczyk et al., 2011; Deng and Hu, 2017), and might due to many factors, i.e., social and cultural norms in the expression of emotion (Hess and Bourgeois, 2010; Rymarczyk et al., 2016). In sum, research regarding the effect of group membership on facial emotional mimicry remains in debate. Thus, further evidence is needed to acquire a better understanding of this issue.

One way to facilitate the understanding of this issue is to investigate the neural underpinnings of the modulation of facial emotional mimicry by group membership. In the present study, we consider the right temporoparietal junction (rTPJ) as a candidate for the neural substrate of the in-group bias in facial emotional mimicry for several reasons. First, rTPJ has been proven to be the neural substrate of online control of coactivation of self and other representations, i.e., self-other overlap (Brass et al., 2005; Donaldson et al., 2015; Martin et al., 2019). Referring to the inclusion of other in the self-representation, the self-other overlap is associated with both behavioral (Lumsden et al., 2014; Maister and Tsakiris, 2016) and facial emotional mimicry (Galinsky et al., 2005; Cooke et al., 2018; Hühnel et al., 2018). In their study, Hühnel et al. (2018) employed a partial inclusion paradigm (Cyberball), where the younger participants were included by the older players but excluded by the young players, to explore the modulation of the in-group bias in facial emotional mimicry by the inclusive behavior of the out-group members (e.g., their elders). The hypothesis was that inclusion by the other could improve overlap of the other and the self (measured by the IOS scale), and this enhanced self-other overlap would thus foster facial emotional mimicry of the others. The result was in line with the hypothesis that younger participants who were partially included by the older players exhibit greater emotional mimicry to the older faces. Hence, it is rational to suppose that excitation of the rTPJ could impel the integration of the others (e.g., out-group) into the self to increase self-other overlap and thus prompt the facial emotional mimicry of out-group expressions. Second, there is research suggesting that rTPJ is one of the neural regions associated with in-group bias in many areas, such as parochial altruism (Baumgartner et al., 2012, 2014; Morese et al., 2016). For example, Baumgartner et al. (2015) found that greater white matter integrity in the TPJ and stronger connectivity between the TPJ and the brain areas involved in the mentalizing network (e.g., dorsomedial prefrontal cortex, dmPFC) were linked to greater impartiality to out-group members. Thus, they decreased intergroup bias. Also, rTPJ has been included as a key part of the neural networks that are related to the modulatory process of emotional mimicry by social contextual factors (i.e., group membership) in previous theoretical models (Wang and Hamilton, 2012; Kraaijenvanger et al., 2017). Taken together, the current study assumes that the rTPJ is a candidate neural area responsible for the modulation of group membership on facial emotional mimicry.

The main purpose of the present study is to use the non-invasive transcranial direct current stimulation (tDCS) to explore whether the rTPJ can modulate facial emotional mimicry. To this end, three different kinds of tDCS stimulations (anodal, cathodal, and sham) are separately performed over the rTPJ of healthy adults. After that, facial EMG response towards happiness and anger of both ethnic in- and out-group models during a facial emotional mimicry task are recorded. Based on the previous studies summarized above, we hypothesize that: (a) there is an in-group bias in facial emotional mimicry in the sham condition; (b) a temporary change of neural activity over the rTPJ modulates in-group bias in facial emotional mimicry. Specifically, excitation of the rTPJ elicits equivalent mimicry of in-group and out-group facial expressions; inhibition of the rTPJ should disrupt the self-other overlap thus leading to greater in-group bias.

## Methods

### Participants

Fifty-one right-handed college students from Renmin University of China (RUC) participated in this study for financial compensation and were randomly assigned into three tDCS groups [anodal group: *n* = 17 (five males, age: 20.41 ± 1.91); cathodal group: *n* = 17 (five males, age: 19.94 ± 1.71); sham group: *n* = 17 (six males, age: 22.58 ± 4.42)]. The sample size was chosen based on previous studies (e.g., Santiesteban et al., 2012; Coll et al., 2017), given that G*Power is unable to conduct power analyses for repeated-measures design with multiple factors. None of the participants reported a history of neurological or psychiatric disorders. Written informed consent was obtained from each participant. This study was approved by the Institutional Review Board of the Department of Psychology, RUC.

### tDCS Protocol

tDCS was performed through a DC-STIMULATOR PLUS (neuroCare Group, Germany). It was delivered through a pair of 35 cm^2^ sponge electrodes, which were soaked in saline. The stimulation site for the rTPJ was the midpoint of CP6 and P6, according to the international 10-20 EEG system, with the reference electrode placed over the left cheek, as in previous studies (Mai et al., 2016). In both the anodal and cathodal conditions, a weak current (1.5 mA) was delivered for 20 min. In the sham condition, the current lasted for only 30 s, though the electrode was in place for 20 min. The fade-in and fade-out time for each condition were both 15 s (Keeser et al., 2011).

### Stimuli and Procedure

After the tDCS protocol, participants were briefly introduced to the experimental procedure. Following prior research, a cover story was given to each participant explaining that this study intended to rate the emotions performed by various models from different ethnics to build a dynamic emotional face database (the rating data was not included in the formal analyses), to make the ethnic salient (van der Schalk et al., 2011; Sachisthal et al., 2016). Electrodes were placed onto the zygomatic major (ZM), corrugator supercilii (CS), and left mastoid to collect the facial EMG data.

**Figure 1:**
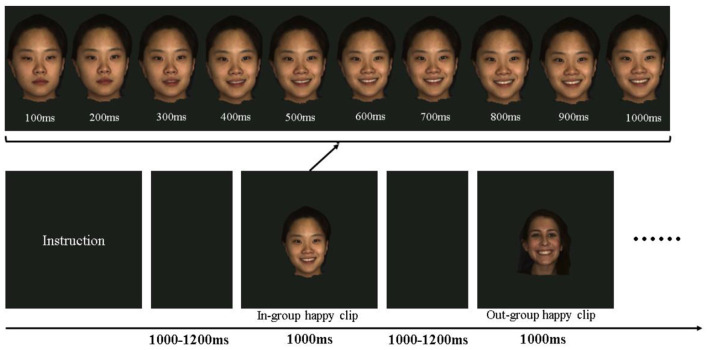
Schematic presentation of the experimental procedure and the materials. In each trial, participants were shown emotional clips at 1,000–1,200 ms intervals. The clips may be a happy or angry emotion performed by an ethnic in-group or an ethnic out-group member. Each clip showed a dynamic presentation of the emotion from a neutral state to the peak emotional state.

As depicted in Figure 1, after an instructional display about the procedure, each participant was then presented with an emotional video clip (depicting happiness or anger) for 1,000 ms in each trial. Previous studies have demonstrated that facial EMG activity is more evidently induced by dynamic facial expressions (e.g., Sato et al., 2008). The order of emotional video clips was randomized for each participant. The intertrial interval varied randomly from 1,000 to 1,200 ms. The emotional clips in the study were obtained from the high-resolution 3D dynamic facial expression database developed by Yin et al. (2008), which contained six basic emotions (happy, angry, fear, sad, disgust, and surprise). The database consists of 58 females and 43 males models, with a variety of ethnic ancestries, including East Asian, White, Black, and Hispanic/Latino. Happy and angry emotional video clips performed by 10 East Asians (ethnic in-group, five males) and 10 White (ethnic out-group, five males) were included as materials, constituting a total of 40 emotional clips. All clips were edited to the same 1,000 ms duration, changing from a neutral expression at the beginning to the full-blown emotional expression by the end (Figure 1). Each stimulus was repeated once in another block.

### Apparatus and Data Analysis

Biopac system EMG (BIOPAC Systems, Inc., Santa Barbara, CA, USA) with a high-pass frequency filter was employed to record the facial EMG activity of the ZM and the CS. Following guidelines of previous research (Fridlund and Cacioppo, 1986), surface Ag/AgCl bipolar electrodes were placed over the ZM and the CS on the left side of the face to measure the facial EMG. The reference electrode was attached to the left mastoid. To reduce the electrode side impedance, the skin over the recording sites was first cleaned with alcohol. The EMG was recorded at 2,048 Hz, with a 28–500 Hz bandpass filter.

The raw data were transferred into EMG signals by calculating the root-mean-squares (RMS) on AcqKnowledge software, version 5.0 (Biopac Systems). The EMG scores were expressed as change in activity in microvolts from the pre-stimulus level, defined as the mean activity during the last second before stimulus onset. Trials with EMG scores of superior three standard deviations from the overall mean value were rejected from subsequent data analysis.

The EMG (ZM and CS) responses were averaged under various conditions. A tDCS (anodal vs. cathodal vs. sham) × Ethnic Group (in-group vs. out-group) × Emotion (happiness vs. anger) × Muscle (ZM vs. CS) four-way mixed ANOVA was conducted on the facial EMG activations, with the tDCS treated as a between-subject variable and Ethnic Group, Emotion, and Muscle treated as within-subject variables. Additionally, in line with prior studies (e.g., Sato et al., 2008; Likowski et al., 2012), one-sample *t*-tests against zero were also conducted to confirm whether the facial emotional mimicry occurred in each condition.

## Results

The mixed ANOVA revealed significant main effects for Emotion (*F*_(1,48)_ = 8.00, *p* = 0.007, *η*^2^ = 0.14), Muscle (*F*_(1,48)_ = 4.54, *p* = 0.038, *η*^2^ = 0.09), and tDCS (*F*_(2,48)_ = 3.34, *p* = 0.04, *η*^2^ = 0.12), which were qualified by significant interactions: Emotion × Muscle (*F*_(1,48)_ = 24.92, *p* < 0.001, *η*^2^ = 0.34); tDCS × Ethnic Group × Muscle (*F*_(2,48)_ = 4.36, *p* = 0.018, *η*^2^ = 0.15); tDCS × Ethnic Group × Emotion (*F*_(2,48)_ = 3.57, *p* = 0.036, *η*^2^ = 0.13); and tDCS × Ethnic Group × Emotion × Muscle, *F*_(2,48)_ = 3.86, *p* = 0.028, *η*^2^ = 0.14. To further investigate the effect of anodal/cathodal tDCS on facial mimicry of in-group/out-group faces, two subsequent tDCS× Ethnic Group × Emotion mixed ANOVAs were conducted on each muscle site.

### ZM Activity

**Figure 2:**
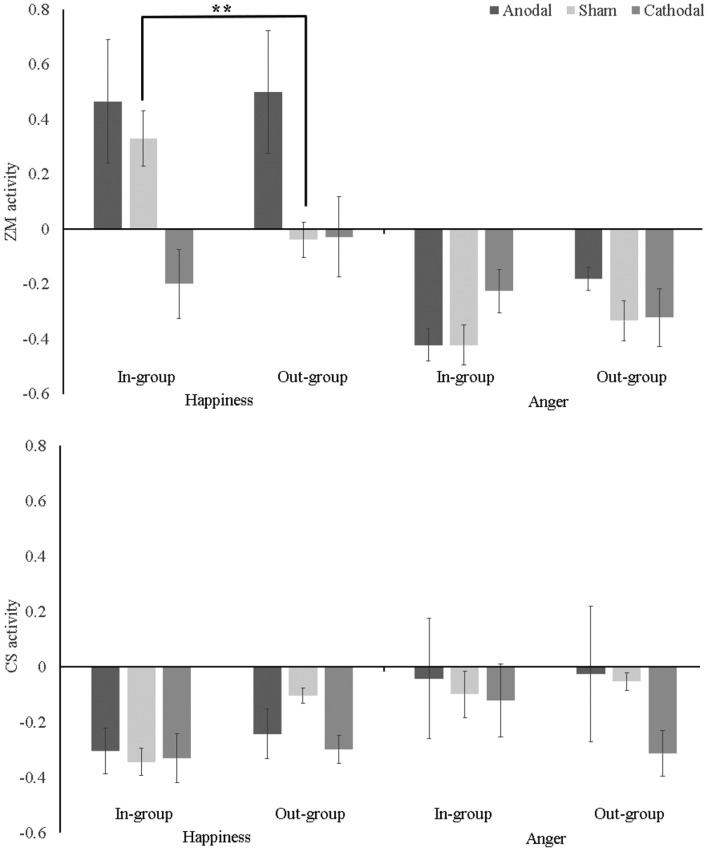
Mean zygomatic major (ZM; upper panel) and corrugator supercilii (CS; lower panel) activity (baseline-corrected) as a function of transcranial direct current stimulation (tDCS), Ethnic Group, and Emotion. Error bar indicates one SE. **p < 0.01.

A tDCS (anodal vs. cathodal vs. sham) × Ethnic Group (in-group vs. out-group) × Emotion (happiness vs. anger) mixed ANOVA on ZM activity revealed significant main effects for tDCS (*F*_(2,48)_ = 2.31, *p* = 0.017, *η*^2^ = 0.16) and Emotion (*F*_(1,48)_ = 31.04, *p* < 0.001, *η*^2^ = 0.39), which were qualified by significant interactions: tDCS × Emotion (*F*_(2,48)_ = 3.40, *p* = 0.041, *η*^2^ = 0.12) and tDCS × Ethnic Group × Emotion (*F*_(2,48)_ = 5.50, *p* = 0.007, *η*^2^ = 0.19). As shown in Figure 2 (upper panel), *post hoc* Bonferroni analyses indicated that ZM response to happiness was greater for in-group than out-group only in the sham condition, 95% CI = [0.096, 0.639], *p* = 0.009. There was no difference between in-group and out-group on ZM activity to happiness, or anger in either anodal or cathodal condition, *p*_s_ > 0.1. Additionally, simple-effect analysis also revealed that: (1) in the sham condition, ZM activation to in-group happiness was stronger than to in-group anger [95% CI (0.430, 1.071), *p* < 0.001], suggesting a mimicry pattern; while ZM activation to out-group happiness was not different from out-group anger [95% CI (−0.024, 0.615), *p* = 0.069], indicating there was no mimicry of out-group happy faces; (2) in the anodal condition, the stronger ZM activities to happiness relative to anger was found for both in-group [95% CI (0.299, 940), *p* < 0.001], and out-group faces [95% CI (0.360, 1.000), *p* < 0.001], suggesting that participants mimicked both in-group and out-group happiness; and (3) in the cathodal condition, the higher ZM activations to happiness than to anger was absent either for in-group [95% CI (−0.295, 0.346), *p* = 0.873] or out-group faces [95% CI (−0.027, 0.613), *p* = 0.072], suggesting participants did not mimic in-group happiness or out-group happiness.

In addition, one-sample *t*-tests against zero found participants showed stronger ZM activation to in-group happiness (*t*_(16)_ = 3.27, *p* = 0.005) but not to out-group happiness (*t*_(16)_ = −0.60, *p* = 0.56) in the sham condition. Furthermore, ZM activation to both in-group (*t*_(16)_ = 2.07, *p* = 0.04) and out-group happiness (*t*_(16)_ = 2.24, *p* = 0.03) were larger than zero. ZM response to angry face in each condition was significantly less than zero, *t*_s_ < −0.1, *p*_s_ < 0.02.

### CS Activity

The mixed ANOVA uncovered a main effect of Emotion (*F*_(1,48)_ = 4.35, *p* = 0.042, *η*^2^ = 0.08), which were qualified by a significant two-way interaction between Ethnic Group and Emotion, *F*_(2,48)_ = 4.85, *p* = 0.033, *η*^2^ = 0.09. *Post hoc* Bonferroni analyses demonstrated the interaction was mainly driven by the fact that CS activation to in-group happiness was weaker than CS activation to both out-group happiness [95% CI (−0.220, −0.002), *p* = 0.045] and in-group anger [95% CI (−0.424, −0.052), *p* = 0.013].

One-sample *t*-tests against zero demonstrated congruent CS activity when seeing happy faces, that was, participants showed below-baseline activation to happiness in each condition, *t*_s_ < −0.3, *p*_s_ < 0.003. However, as shown in Figure 2 (lower panel), when seeing angry faces, CS activation was not larger than zero in either condition, suggesting participants did not mimic either in-group or out-group anger in any tDCS conditions, *p*_s_ > 0.2, expect that CS to out-group anger was less than zero in the cathodal condition, *t*_(1,16)_ = −3.78, *p* = 0.002.

## Discussion

In-group bias occurs in many areas, including facial emotional mimicry, a tendency to automatically match the observed facial expressions to better acknowledge others’ emotional state or foster social relation. The present study is designed to explore the role of rTPJ in the modulation of facial emotional mimicry by group membership. Using tDCS to temporarily change the cortical activity over the rTPJ, we have demonstrated that rTPJ can alter the in-group (relative to out-group) advantage in facial emotional mimicry. Specifically, we confirm the in-group bias in facial emotional mimicry in the sham condition, which is in line with prior research (Weisbuch and Ambady, 2008; Deng, 2018; de Klerk et al., 2019). We further reveal that this in-group bias in facial emotional mimicry is regulated by the temporal change of neural excitability over the rTPJ.

### In-Group Bias in Facial Emotional Mimicry

The present study recorded the EMG activity from ZM and CS muscle regions as the index of happy and angry facial mimicry, respectively. This is consistent with most prior research (Sato et al., 2008; van der Schalk et al., 2011; Rymarczyk et al., 2016; Deng and Hu, 2017), while there are other studies suggested facial emotional mimicry should be indicated as a certain pattern of EMG activity that is related to relevant facial muscles, for example, happy mimicry is indexed as the contraction of ZM and the relaxation of CS (Hess et al., 2017; Hühnel et al., 2018). We employed a single muscle measurement based on several reasons. First, previous studies using different methods obtained the same results (e.g., Bourgeois and Hess, 2008; van der Schalk et al., 2011), although there is no research to date directly compare the results induced by these two methods. Second, it is hard to determine facial muscles corresponding to the imitated emotion, for example, happy mimicry does not always induce relaxation of CS (e.g., Seibt et al., 2015). This might be the reason that mimicry index derived from several facial muscles suffers from low reliability (Hess et al., 2017). It should be noted we do not disapprove of the measurement of several facial muscles relating to mimicked expressions. More efforts should be made to determine the specified muscle group corresponding to the imitated emotion.

The findings in the sham condition reveal an in-group bias in facial emotional mimicry, especially for happiness. Although both in-group and out-group happy faces induce congruent reactions (a contraction of ZM and relaxation of CS relative to baseline), ZM responses to in-group happiness are stronger than that to out-group happiness, which is contrary to Bourgeois and Hess (2008) who demonstrated that mimicry of happiness is independent of group membership, since that happiness is a “low-cost” emotion. However, is the mimicry of happiness prevalent regardless of any social contextual factors? This answer should be negative. For instance, Carr et al. (2014) showed that high-power people exhibit happy mimicry toward low-power people but not toward other high-power people. Furthermore, there is surprisingly no sign of happy mimicry in van der Schalk et al. (2011) study, regardless of the measurement (EMG in Study 1 and Facial Action Coding System (FACS) in Study 2) or the manipulation of group membership (a subjective category in Study 1 and ethnicity in Study 2). It is therefore more practical to say that happiness is a conditional “low-cost” emotion that is modulated by some contextual factors in facial emotional mimicry. The selective mimicry of in-group happiness in our study can also be elucidated by Weisbuch and Ambady (2008), who advanced that people decide to mimic the observed emotional expression or not based on its meaning or function, which was found to be further affected by whether or not the emotional perceiver and emotional sender share group membership (Elfenbein and Ambady, 2002; Hareli and Hess, 2012). According to Weisbuch and Ambady (2008), for example, a happy face may signal “safety.” However, when it appears on an out-group member’s face, it signals strength and dominance of the out-group relative to the in-group and the self, and thus the out-group happy faces should elicit less mimicry than the in-group happy faces, as demonstrated in the present study.

In terms of anger, EMG activities in the sham condition display an incongruent reaction. Although relaxations of ZM for angry displays are found, the EMG activity from CS muscle is not different from the baseline, either for in-group or out-group anger. This means participants in the sham condition did not mimic angry faces, which is consistent with prior research (e.g., Weyers et al., 2006). Anger is a signal of conflict for both the in-group and the out-group, with the slight difference that the out-group anger should index relatively stronger conflict than in-group anger (Weisbuch and Ambady, 2008), which may be partially attributable to an in-group bias in anger mimicry, presented in some previous studies (e.g., van der Schalk et al., 2011) but not in others (e.g., Sachisthal et al., 2016). Also, social and cultural norms in the expression of negative emotions (i.e., anger) may be responsible for the current and many previous studies that did not find mimicry of angry faces (Hess and Bourgeois, 2010; Rymarczyk et al., 2016; Deng and Hu, 2017). People from China (as in the current study), a typical collective culture that highly values the interdependence with others, tend to define their self based on the connection with others [termed as relationalism by Hwang (2000)] and suppress the expression of negative emotions to keep a harmonious personal relationship (Chiang, 2012; Wei et al., 2013). Future studies are expected to employ between-culture comparison to probe this issue.

Overall, the current study demonstrates group membership can affect facial emotional mimicry, supports the Emotional Mimicry in-Context view (Hess and Fischer, 2013, 2014). Together with prior research employing Western samples, this study provides evidence that the modulatory effect of group membership on facial emotional mimicry occurs cross-culturally. However, there are also cultural variances in the expression of negative emotions, which might be responsible for the inconsistent results reported in previous studies. Unlike Western samples, participants from China (the most typical collective culture) generally do not show mimicry of others’ angry faces, either for in-group or out-group members.

### rTPJ Modulates In-Group Bias in Facial Emotional Mimicry

Since there is no obvious mimicry-related EMG activation in response to either in-group or out-group angry faces in any of the three tDCS conditions, we focus our discussion of the rTPJ majorly on happy mimicry.

The current study is likely the first one to use tDCS to test the neural substrates of the modulation of facial emotional mimicry by group membership. Unlike the fMRI and fNIRS, tDCS provides a causal relationship between the rTPJ and the modulation of facial emotional mimicry by group membership, expanding the understanding of the neural underpinnings associated with facial emotional mimicry in a different social context. In line with our prediction, excitation of the rTPJ does evoke greater mimicry of out-group happiness, compared to the sham condition that displays no mimicry-related EMG response to out-group happy faces. More importantly, there is no difference between the EMG activation induced by in-group and out-group happiness in the anodal tDCS condition, suggesting that excitation of the rTPJ yields equivalent happy mimicry independent of an ethnic group membership. A possible explanation is that excitation of the rTPJ makes people more likely to inhibit their egocentric-perspective (Martin et al., 2019) and blend the others into self-representation (“coactivation of the self and the other” proposed by Santiesteban et al., 2012), and this enhanced self-other overlap then fosters greater mimicry of the others (Cooke et al., 2018; Hühnel et al., 2018). Specifically, in the current study, participants received anodal stimulation of the rTPJ were more likely to integrate the representations of the in-group and the out-group, and thus their mimicry of in-group and out-group faces is indiscriminatory. The result of the cathodal tDCS condition seems to support this idea. Since the rTPJ is predisposed to greater representation of the in-group members relative to the out-group members, which is accountable for the intergroup bias revealed in previous works (e.g., Baumgartner et al., 2012, 2015), diminished cortical activity over the rTPJ should disrupt this kind of overall ingroup-advantage in mental representation. Consistent with this explanation, a recent study (Hühnel et al., 2018) has suggested that an increased level of self-other overlap is associated with higher mimicry of the out-group. Conclusively, this study goes beyond the previous studies (e.g., Baumgartner et al., 2014) and suggests that enhanced rTPJ activity is associated with the reduced inter-group bias in facial emotional mimicry as well.

One might speculate that our findings could also be explained by the fact that rTPJ is a key brain area relating to facial emotional mimicry. However, there is no research to date showing the excitation of rTPJ during mimicry of facial expressions (Lee et al., 2006; Likowski et al., 2012). Additionally, mimicry of in-group happiness should be stronger in the anodal condition than in the sham condition, provided that enhanced rTPJ activity is associated with stronger mimicry. However, the ZM response to in-group happy faces in the anodal condition is not different from that of the sham condition.

The role of rTPJ in on-line control of self and other representations (i.e., the biasing of processing toward either the self or the other when task demands cause both the self and the other to be represented—quoted from Santiesteban et al., 2012) has been substantially investigated in prior research. Some studies found the role of rTPJ was to distinguish self and other representations, by showing anodal tDCS over rTPJ increased the performance in imitation inhibition task, which required participants inhibit other representation and enhance self-representation (Sowden and Catmur, 2015; Nobusako et al., 2017; Duffy et al., 2019). Other studies found the role of rTPJ was to inhibit or shift self-representation to other representation (Wang et al., 2016; Martin et al., 2019). A tentative assumption is that the role of rTPJ is flexible in self-other representation, either self-other overlap or self-other distinction, dependent on the task demands (Hogeveen et al., 2014). The present study tends to agree with the idea that anodal stimulation of the rTPJ improved self-other overlap (shared representations of in-group and out-group), which is in line previous studies (Lombardo et al., 2010; Santiesteban et al., 2012; Santiesteban, 2014). Besides, there is also research that suggests that rTPJ is involved in the mental attribution of both self and others (Frith and Frith, 2003; Adolphs, 2009). Hence, together with Santiesteban et al. (2012), the current study indicates rTPJ is recruited in social cognition (e.g., facial emotional mimicry) where on-line control of coactivated self and other representation is essential since facial emotional mimicry fosters social bonds (Hess and Fischer, 2017).

The current outcomes should have implications for the reduction of intergroup bias and facilitation to inter-group communication. As shown in the present study and other previous studies, the change of the mental representation of in-group and out-group, induced by either exciting the rTPJ or by inclusion from an out-group member (Hühnel et al., 2018), facilitates mimicry of out-group faces and thus eliminates the inter-group bias in facial emotional mimicry. The in-group bias in facial emotional mimicry does enhance the ingroup cohesion but also exaggerates the out-group negativity, which is harmful to intergroup communications (Hess and Fischer, 2017). Mimicry reduces racial prejudice and increases liking and closeness toward out-groups (Stel and Vonk, 2010; Inzlicht et al., 2012). Hence, finding ways to erase the in-group bias in facial emotional mimicry is of particular significance in social interaction. This study provides a neural causal interpretation for the improvement of out-group facial mimicry by increasing ingroup-outgroup overlap.

## Limitations

Several limitations of the current study should be acknowledged. First, the relatively short duration of the emotional clips used in our study might interfere with the results. However, previous studies have also demonstrated facial emotional mimicry using dynamic emotional expressions lasting about 1,000 ms (e.g., Achaibou et al., 2008; Deng and Hu, 2017). Second, though previous studies have already confirmed that the tDCS could change the excitability of the cortex (e.g., Jaberzadeh and Zoghi, 2013), the shortage of its lower spatial precision makes it difficult to confirm the functions of the rTPJ subregions. Thus, future studies are encouraged to combine other neuroimaging techniques, such as fMRI and fNIRS, to investigate the role of those subregions of rTPJ in facial emotional mimicry. A third limitation of the current study is the lack of measurement of the self-other overlap that has been used in prior research (e.g., Cooke et al., 2018; Hühnel et al., 2018). The reason for this is the concern that the inclusion of such measurement during the procedure could evoke participants’ explicit attention to this self-other overlap and thus interfere with their natural facial emotional mimicry reactions.

## Conclusion

In conclusion, this study demonstrates there is an in-group advantage in facial emotional mimicry, specifically, a happy expression performed by an in-group member is mimicked to a stronger degree than a happy face of an out-group member. Furthermore, this in-group bias is modulated by the cortical excitability over the rTPJ. The underlying mechanism may be that exciting the rTPJ promotes the self-other overlap between mental representations of the in-group and out-group, and thus erases the in-group bias by increasing the mimicry of out-group faces. This study highlights the role of rTPJ in on-line control of co-activated self and other representations in social cognition.

## Data Availability Statement

The datasets generated for this study are available on request to the corresponding authors.

## Ethics Statement

The studies involving human participants were reviewed and approved by Institutional Review Board of Department of Psychology, RUC. The patients/participants provided their written informed consent to participate in this study.

## Author Contributions

SP and PH designed the study. SP collected, analyzed the data, and wrote the draft. SP, BK, and PH revised the draft.

## Conflict of Interest

The authors declare that the research was conducted in the absence of any commercial or financial relationships that could be construed as a potential conflict of interest.
